# Translating Science Into Business Innovation: The Case of Open Food and Nutrition Data Hackathons

**DOI:** 10.3389/fnut.2018.00096

**Published:** 2018-10-22

**Authors:** Christopher Tucci, Gianluigi Viscusi, Heidi Gautschi

**Affiliations:** ^1^Chair of Corporate Strategy and Innovation, College of Management, École Polytechnique Fédérale de Lausanne, Lausanne, Switzerland; ^2^Haute École Pédagogique du Canton de Vaud, Lausanne, Switzerland

**Keywords:** business innovation, open data, hackathons, nutrition data, open innovation

## Abstract

In this article, we explore the use of hackathons and open data in corporations' open innovation portfolios, addressing a new way for companies to tap into the creativity and innovation of early-stage startup culture, in this case applied to the food and nutrition sector. We study the first Open Food Data Hackdays, held on 10–11 February 2017 in Lausanne and Zurich. The aim of the overall project that the Hackdays event was part of was to use open food and nutrition data as a driver for business innovation. We see hackathons as a new tool in the innovation manager's toolkit, a kind of live crowdsourcing exercise that goes beyond traditional ideation and develops a variety of prototypes and new ideas for business innovation. Companies then have the option of working with entrepreneurs and taking some the ideas forward.

## Introduction

The word “digital” has moved from the technical domain to everyday life, encompassing not only information systems and their output, but also the way we frame different societal issues. This process involves not only access to digital technologies but also influences services and products in both the private and public sector. The digital transformation of industry is also enabling and amplifying firms' *open innovation* processes, in which companies seek new ideas and new technologies from the outside, while simultaneously attempting to monetize or exploit internal ideas that do not fit the company's business model. Firms often use an open innovation approach to help make a transition from their traditional markets to new ones that in this case take advantage of information technologies by creating new products, developing new services, and changing the product/service mix or other business model components relative to their past activities.

In this article, we explore the use of *hackathons* (challenges in the form of a one- or more day event where a crowd of people meet to code and develop new applications, services, etc.) and *open data* (data whose access is available to anyone) in corporations' open innovation portfolios, addressing a new way for companies to tap into the creativity and innovation of early-stage startup culture, in this case applied to the food and nutrition sector. This sector, like many others, is in the process of several scientific, technological, and even societal transformations, which include a better understanding of personalized nutrition, digital transformation of the food supply chain, and increased demand for health and nutritional transparency (1–5). This is leading to increased entrepreneurial and innovation activity in the sector based in many cases on data repositories that are open to anyone, in many cases via structured protocols called Application Programming Interfaces, or APIs, such as datasets made available by departments of agriculture in different countries (e.g., FarmPlenty described at https://www.data.gov/food/ taking advantage of USDA open data on crops) or built upon APIs from the companies owning the data, or even collected via crowdsourcing or scraping. Getting input from the crowd via hackathons can be an interesting and cost-effective way to supplement a company's open innovation activities, as discussed further below.

## Background and motivations

### Challenges of digitization

“Digitalization” has been identified as a sociotechnical process (i.e., a process involving both people and technology) that exploits the encoding of analog information in digital format (the technical process of “digitizing”) in a larger and systematic fashion and rendering digital technologies infrastructural (6, 7). The resulting digital artifacts can then be stored and used in a distributed fashion, can be edited and shared, and maybe made more interactive (8). The way companies think about innovation could be moving from a more “controlling” process emphasizing breaking the problem or system into smaller, less interdependent pieces to something more “generative” (9–11), where generativity is considered “*a technology's overall capacity to produce unprompted change driven by large, varied, and uncoordinated audiences*” [12, p. 1980]. Digitalization may be used to improve business processes [13, p. 224] encourage open innovation (14) or crowdsourcing (15). In the context of **nutrition and food**, food data represent a key resource for innovation in different areas, starting from traditional agriculture (16) to new trends such as 3D food (17) and the emergence of the field of Human Food Interaction (HFI), paving the way to understanding how companies capture the potential benefits (18, 19). No matter which industry, many public and private organizations have identified a need to explore new business models (20) for creating and capturing value from their activities (21) and digitalization is reinforcing this imperative.

### Innovation in open data

Open data have attracted growing interest in academia as well as among practitioners, especially in the public sector, due partly to the diffusion of open government data initiatives and investments, with a consequent set of benefits (such as increased transparency, creation of a new markets, and improved policies) and limitations, such as the need for organizational and institutional settings for value creation from open data, privacy, and security harm [cf. 22, 23]. However, the use of open data to provide value (either public, social, or economic) can only occur in an “ecosystem” of actors involving businesses, suppliers, intermediaries, developers, data aggregators, and other “complementors” (24, 25), possibly oriented toward new *infomediary* business models between open data providers and users (26), where an infomediary originally described a broker that enables users to deal with large volumes of data to obtain the right information for their needs while safeguarding privacy [26, p. 2]. Over time, this concept has broadened to mean the handling of information between information providers and consumers and a literature has developed [e.g., 27–33] on business models for open data applications. Based on the use of open data by 178 U.S. firms, Magalhaes and Roseira [31, p. 9] developed twelve atomic business models, including *single purpose apps, interactive apps, data platforms, open data portals*, and *business intelligence*. However, in general, open data and open government data have mainly received attention in the policy and technical literatures, with little connection to the management and open innovation literature (32, 33). These initiatives could potentially be key drivers for developing new business models with open data in private companies.

Related to this, *hackathons* have received specific attention, or as in the case of “datathons” or hackathons for data analysis (34, 35), to carry out data scientists' tasks related to data management, data quality, data analytics, data visualization, etc. (36). Nevertheless, as pointed out by Kitsios et al. (37), although hackathons are considered a key activity by participants to open data ecosystems to identify new business models and develop entrepreneurship, none of their six cases “realize[d] the impact of open data in economic growth but only in social growth.” Consequently, hackathons also represent a key activity for identifying business models related to new applications of open data for many areas, including the food and nutrition sector. In what follows, we present a case study aiming to bring these different themes together.

## Case study: the “open food data hackdays” hackathon

The first Open Food Data Hackdays were held on February 10 and 11, 2017, at two venues in Switzerland: The Ecole Polytechnique Fédérale in Lausanne (EPFL) and the School of Art in Zurich. 192 people signed up to attend the event in Lausanne and 141 in Zurich. The hackdays were organized by Opendata.ch and funded by Engagement Migros, a development fund of the Migros Foundation. The underlying purpose of this event was to promote the use of open food and nutrition data for businesses innovation. The Hackdays is part of a 3-year project that “aims to build a publicly available base of nutrition data, to create new innovative and value adding solutions, and to further develop the use of open data for entrepreneurial purposes” (38). Given that the aim of the overall project that the hackdays event was part of, to use open food and nutrition data as a driver for business innovation, the organizers invited individuals and groups to propose projects (frequently called “challenges” in hackathons) for hackday participants to work on that could become viable businesses. A team of three researchers from the College of Management at EPFL attended the hackdays. Two of the researchers observed the Zurich event and one researcher observed the event in Lausanne.

The Open Food Data Hackdays event was similarly organized in both venues. On the morning of the 10th of February, the organizers in both venues presented the event to the participants. Teams would have 24 h to work on a project and present a prototype at the end of the event. A jury made up of members of the organizing and funding teams would choose a small number of projects to be incubated (provided office space, modest funding, and interaction with the company) and coached over the following 2–3 months. The research team from EPFL also gave their input in the form of evaluation criteria and their evaluations of the projects. After the initial presentation, the projects selected by the organizers prior to the event were pitched to the participants. The floor was then opened to additional projects. Participants then self-selected into teams of 5–10 people (average: 9) to work on projects of interest that were pitched. Crucially, each pitch included a problem to solve, or a goal to reach within the time frame of the Hackdays. Hackathons are generally organized with both a high-level theme and specific challenges and the Hackdays was no exception. All the projects revolved around using open food and nutrition data sets such as Migros Nutrition Facts, or Swiss food waste data, or Swiss government data on fresh product nutrition; and/or creating open food and nutrition data sets, such as the carbon footprints of every type of domestic and imported food. Open food and nutrition data includes everything from information on food labels to geo-localization data of farmers markets. The projects were equally diverse and included everything from coding the chemical makeup of beer to mapping local farmers to finding innovative ways of encouraging people to eat more balanced meals. Some challenges presented were already businesses and others were barely in the ideation phase. All intellectual property remained with the teams, who could decide whether to take the ideas further on their own or with the sponsor's support.

The winning projects announced a few days after the event reflect the diversity of opportunities presented by open food and nutrition data. Open receipts (https://hack.opendata.ch/project/74), Nutrimenu (https://hack.opendata.ch/project/68), and Jarvis the Nutritionist (https://hack.opendata.ch/project/60) were selected from Lausanne. Open receipts seeks to transform your supermarket receipts into “actionable data” that would give you information about the calories in the food you purchased and allergens, for example. Nutrimenu was already collaborating with the city of Lausanne prior to the Hackdays. Their goal by attending the Hackdays was to continue improving their product. Nutrimenu is an application that helps you create healthier and tastier meals. It won the 2017 award for Swiss health enterprises. Meat Story (https://hack.opendata.ch/project/73) and Foodimmune (https://hack.opendata.ch/project/79) came out of Zurich. Meat Story seeks to make the meat you buy traceable from farm to fork through the use of a mobile app and possibly QR codes. Foodimmune leverages the medicinal properties of food to help you stay healthy. These five projects are being followed by the team of researchers from the College of Management. Data is being collected to further elucidate the impact of open food and nutrition data on business innovation. As data collection is ongoing, the results have not been included here.

Who were the participants? After the Hackdays event, a survey was sent out to the participants and we received 44 responses (roughly 13% of those signing up and 20% of those attending). The majority of the participants were between 19 and 45 years old (19–27: 29.5%; 28–35: 27.3%; 36–45: 31.8%) and 70.5% of the respondents identified as male. 86.4% had received either a bachelor, master, doctoral, or law degree. Most respondents studied computer science and engineering (75% combined). It is interesting that despite the open food and nutrition data theme so few respondents had social science or life science degrees^1^. We were interested in trying to better understand the motivations of the participants and asked a series of questions using a seven-point Likert scale (1 = strongly disagree to 7 = strongly agree) to ascertain what motivates people to attend hackathons. Three motivators stood out: attending the hackathon was a way to enhance skills (68.2% chose 5 or above) and participating in the hackathon allowed participants to explore their strengths and limitations (72.8% chose 5 or above); most participants (77.3% chose 5 and above) attended the hackathon to learn about open food and nutrition data challenges. Given the sponsor and the overall goal of this event, it should come as little surprise that most survey responders cared about the open data movement (81.3% chose 5 or above with 37.2% choosing 7—strongly agree) and saw the hackday event as a way of participating in the open data cause (65% chose 5 or above). Forty six percent of respondents strongly agreed that it is important to participate in initiatives like the hackdays event. While the structure of the hackdays could be seen as inducing competition, survey respondents did not participate in the hackdays to beat others at solving a problem (33% strongly disagreed, 28.6% chose 2, and 11.9% chose 3). It is unclear whether this would be the case for hackathons in general, or was specific to the Open Food Data Hackdays event.

In terms of cost, while we do not have access to detailed cost information from Migros Engagement, we can estimate that the food, space, and overall organization / facilitation would run about $120,000 and the incubation would cost roughly $20,000. Thus for roughly half the cost of one R&D personnel for 1 year, the company generated a dozen credible prototypes, hundreds of ideas, and the option of further developing new lines of business or insights into consumer behavior.

## Discussion and conclusion

We see hackathons as a new tool in the innovation manager's toolkit, a kind of live crowdsourcing exercise that goes beyond traditional ideation. Of course, the size of the crowd may be much more limited in a hackathon relative to a large, public crowdsourcing challenge; on the other hand, the level of interactivity and the parallel structure of the teamwork can lead to interesting solutions, communication of those solutions via prototyping, and inspiration for the sponsor to take some of the ideas further in a short period of time (see Table 1, showing the winning teams feedback, providing an understanding of the winner ideas and how these could profitably use the open data repository, among other issues). In addition, sponsor organizations may gain insight into aspects of the challenge that resonate with external innovation teams, input that is often hard to come by via other means such as focus groups or external consultants. Regarding digital innovation in particular, there are several aspects of the hackathon approach that corporate innovation managers may want to consider as part of a digital transformation. The first is the ability (and decreasing cost) to attract and manage crowds due to the IT project and collaboration tools that are currently available. These tools are helpful in organizing a team's strategy for developing a protoype and working together on the same documents, for example. The second is the ability to gain insight into possible strategic digital innovations by examining the overall portfolio of ideas emanating from the hackathon, in this particular case, how the open data was being proposed to be used and analyzed by the teams. We speculate that such insights could be interesting in developing new business models or opening up new value networks and entirely new markets built upon the key competences in the main business. And finally and even more speculatively, hackathons could be a possible recruiting tool for digital natives as they interact with the sponsor organization and gain a positive impression of the sponsor's open-mindedness.

**Table 1:** Feedback from winning teams.

**Team**	**Vision**	**Goals**	**Accomplishments**	**Support**	**Next steps**
Jarvis	Chatbot is a new way to interact with products	Build a recommender system Build a chatbot interface	Scraped recipes from allrecipes Built chatbot prototype Information gathering about chatbot and recommender system	Financing Meeting with Hannes Gassert Meeting with Thomas Rippel	Complete recommender system prototype Complete chatbot Test bot with real users Launch strategy (March 2018)
Open receipts	Unlock the data stored in receipts and turning into insights for consumers	Develop a minimal viable product (MVP)	Developed a minimal viable product Identified consumer needs Developed a business use case	Coaching (could have been more timely) Financing	Test current product and further develop its functionality
Food immune	Mobile application inspire healthy eating based on herbs and local ingredients	Limits of the concept Develop, design the app Test prototype Fully functioning downloadable MVP	Narrowed down concept Created website Tested early prototype Reiterated design Market research Further refined product	Able to attend global summit Coaching Financing	Rethinking project
Nutrimenu	Extend client base	Assess demand Adapt the product to new customers Present the product at conferences & expos Look for funding	Sent a survey to 6,000 restaurants Met with 12+ catering companies Market research for product in Swiss German cities Presented product at 6 venues Applied for funding, support & collaboration	Financing Helped with outreach to German speaking cities Coaching Offered visibility and credibility	Translate product in German Continue seeking new clients Continue developing the product
MeatStory	Trace origin of meat	Build a MVP Test product on real customers Gather feedback and reiterate	Press releases in 2 newspapers Access to data Meetings with potential partners Prototype design sessions Market research Presentation at Opendata event Developed two versions of product	Hackdays Inspiring network Regular calls and meetings financing	Marketing Testing

To conclude, companies in the food and nutrition sector are quite adept at product and process innovation, especially product extensions, operational excellence, and supply chain optimization. However, it is less obvious how to gain insight into more radical opportunities for future markets and business models by observing and interacting with entrepreneurs and digital natives without making large investments in hiring, and, even then, hiring in specific areas commit the sponsor to a less flexible future course. In this case, the theme of open data also attracted participants who were in many cases philosophically aligned with the social impact of the theme, thus enabling the sponsor to motivate and attract a large and enthusiastic crowd for a short period of time at very low cost. Hackathons may therefore have an important role to play in corporate open innovation and crowdsourcing activities, especially in those sectors in which it might be more difficult, expensive, or undesirable to engage crowds of younger, tech-savvy people for long periods of time.

## Ethics statement

We hereby confirm that for this type of study, formal consent is not required. This is in line with the Swiss Federal Act on Research involving Human Beings (Human Research Act, HRA, 2011), especially considering Art.2 that defines the boundary of its application to research concerning human diseases and concerning the structure and function of the human body, which involves: (a) persons, (b) deceased persons, (c) embryos and fetuses, (d) biological material, (e) health-related personal data. It does not apply to research which involves: (a) IVF embryos in accordance with the Stem Cell Research Act of 19 December 2003, (b) anonymized biological material, (c) anonymously collected or anonymized health-related data. Also, it complies with other international legislations such as, e.g., the United States Electronic Code of Federal Regulations eCFR, especially §46.104 Exempt research, specifically, (2): Research that only includes interactions involving educational tests (cognitive, diagnostic, aptitude, achievement), survey procedures, interview procedures, or observation of public behavior (including visual or auditory recording) if at least one of the following criteria is met: (i) The information obtained is recorded by the investigator in such a manner that the identity of the human subjects cannot readily be ascertained, directly, or through identifiers linked to the subjects; (ii) Any disclosure of the human subjects' responses outside the research would not reasonably place the subjects at risk of criminal or civil liability or be damaging to the subjects' financial standing, employability, educational advancement, or reputation; or (iii) The information obtained is recorded by the investigator in such a manner that the identity of the human subjects can readily be ascertained, directly or through identifiers linked to the subjects, and an IRB conducts a limited IRB review to make the determination required by §46.111 (a) (7).

## Author contributions

CT, HG, and GV designed the study. GV contributed to the literature search. HG and GV contributed to the case studies. CT, HG, and GV drafted the manuscript. CT revised and finalized the manuscript.

### Conflict of interest statement

The authors declare that the research was conducted in the absence of any commercial or financial relationships that could be construed as a potential conflict of interest. However, we did receive a small grant as part of this study from a non-profit organization that also granted us access to the hackathon participants.

## Footnotes

^1^This should be considered a limitation of the study. While it is clear that the participants generated many ideas and prototypes related to food and nutrition data, one would need to filter and develop much further with life science experts any ideas that made health claims or that involved personalized medicine.
